# Information and Communications Technology in Dentistry: an informative and educational approach for patients with fixed orthodontic appliances

**DOI:** 10.1590/2177-6709.27.3.e22spe3

**Published:** 2022-07-04

**Authors:** Marina Araújo Leite ASSIS, Lílian Dayse Fróes TAVARES, Aline Pereira BERNARDINO, Breno Amaral ROCHA, Lucas Guimarães ABREU, Dauro Douglas OLIVEIRA, Matheus Melo PITHON, Rodrigo Villamarim SOARES

**Affiliations:** 1 Pontifícia Universidade Católica de Minas Gerais, Departamento de Odontologia (Belo Horizonte/MG, Brazil).; 2 Universidade Federal de Minas Gerais, Departamento de Odontopediatria e Ortodontia (Belo Horizonte/MG, Brazil).; 3 Universidade Estadual do Sudoeste da Bahia, Departamento de Saúde I (Jequié/BA, Brazil).

**Keywords:** Information technology, Orthodontics, Fixed orthodontic appliances

## Abstract

**Objective:**

To develop and make available, at no cost to the user, Information and Communications Technology (ICT) tools for Dentistry, providing dental information and advice geared toward patients undergoing orthodontic treatment with fixed appliances.

**Material and Methods:**

A Dentistry-based content that contemplated information and advice concerning orthodontic treatment with fixed appliances was elaborated. The materials, which included instructions on oral hygiene and treatment strategies when faced with possible complications, were evaluated and validated by specialists, whose assessments reached a 85% approval. From the validated content, products using four distinct ICT tools were formulated.

**Results:**

The following technological products were developed: a program for community radios, three blog posts, four educational and informative videos, and a smartphone application - using texts, as well as images and videos. These ICT tools, geared toward patients wearing fixed orthodontic appliances, were made available by internet at no cost to the user, and the number of accesses is already expressive.

**Conclusion:**

These technological-scientific tools, developed and provided freely to the population, can aid patients during their treatment with fixed orthodontic appliances, contributing to the dissemination of reliable information, and clarifying doubts that may arise during orthodontic therapy. These free ICT tools serve to facilitate access to scientific knowledge, thereby favoring social inclusion, bearing in mind that this educational and informative material was offered in a simple and accessible manner to the general population.

## RESUMO

**Objetivos:**

Desenvolver e disponibilizar, gratuitamente, ferramentas de tecnologia da informação e comunicação (TIC) para a Odontologia, abordando informações e orientações direcionadas a pacientes em tratamento ortodôntico com aparelho fixo.

**Material e Métodos:**

Foi elaborado um conteúdo que contemplou informações e orientações concernentes ao tratamento ortodôntico com aparelho fixo. O material, que abrangeu instruções sobre higiene bucal e sobre abordagens diante de possíveis intercorrências, foi avaliado e validado por especialistas. A partir do conteúdo validado, foram elaborados produtos utilizando quatro ferramentas distintas de TIC.

**Resultados:**

Foram desenvolvidos os seguintes produtos tecnológicos: um programa para rádios comunitárias, três postagens para *blog*, quatro vídeos educacionais e informativos, e um aplicativo para *smartphones*, utilizando não apenas textos, mas também imagens e vídeos. Essas ferramentas de TIC direcionadas a pacientes fazendo uso de aparelho ortodôntico fixo foram disponibilizadas gratuitamente via internet, e já contam com expressivo número de acessos.

**Conclusões:**

As ferramentas técnico-científicas, desenvolvidas e fornecidas livremente à população, podem auxiliar pacientes durante o tratamento com aparelho ortodôntico fixo, contribuir para disseminar informações confiáveis e esclarecer dúvidas que surgem durante a terapia ortodôntica. Essas ferramentas gratuitas de TIC facilitam o acesso ao conhecimento científico e, consequentemente, favorecem a inserção social, tendo em vista que esse material educacional e informativo foi ofertado de maneira simples e acessível à população.

## INTRODUCTION

Recent decades have witnessed a significant increase in the use of new technologies that have transformed the personal and professional lives of millions of people and influenced culture, education, and international business.^1^ ^,^ ^2^ In this scenario, the use of Information and Communications Technology (ICT) has allowed expressive advances in the health services area through the use of virtual tools available on the internet.^3^ ^,^ ^4^

This versatility provided by ICT allows for healthcare advice to be provided through a wider variety of formatting modes (images, text, video, audio), which can favor the availability and accessibility of information. In this context, and resisting to decades of change, radio programs continue to be a widely renowned communication strategy for social, cultural, and healthcare development of many communities.^5^ With the advent of the internet, the blogs have become another important and popular tool, which configures as a mean through which information about health can be disseminated in a wide and fast manner.^6^ Concerning videos, there is evidence that their use is effective as an auxiliary tool in education and in health promotion.^7^ Smartphone applications can present images, videos, audios, and text resources, providing a greater patient/professional interaction, and facilitating the access to medical advice and to important care and treatment guidelines, diagnosis, and patient follow-up.^8^ ^,^ ^9^

Dental professionals need to instruct their patients, help them to understand the importance of the recommendations given, and motivate them to maintain treatment adherence. In practice, this need has proven to be particularly difficult in Orthodontics, due mainly to the young age of most patients, and to the long period of time required for treatment.^10^ For this reason, there is a growing interest in the use of new technologies and protocols to motivate orthodontic patients, with some studies demonstrating the efficacy of tools, such as short message service (SMS), in the reduction of pain levels, treatment time, negative effects in the daily routine of patients and in improvements in dental care.^11^ ^,^ ^12^ The provision of content about orthodontic therapy, through a wide range of ICTs, can promote an enrichment in the instructions inherent to the adopted protocols and in the quality of treatment.^13^ In addition, the use of such technologies can benefit the motivation and adherence to treatment by the patient, in much the same way as it can reduce urgent orthodontic dental appointments, making them much more well-organized.^1^

However, the variety of content related to Dentistry disseminated on the internet present a questionable quality of information, which is often unreliable.^14^ ^,^ ^15^ Faced with this reality, the present study sought to develop and make available, at no cost to the user, specific ICT tools for Dentistry, information on orthodontic treatment, and advice geared toward patients undergoing orthodontic treatment with fixed appliances. To achieve this goal, a specific content was created and validated, with the aim of promoting self-care and contributing to advances in treatment integrity.

## MATERIAL AND METHODS

Among a wide range of information and guidelines related to orthodontic treatment with fixed appliances - considering its pertinence, the greater amplitude provided, and thinking about the different steps of the process -, a content was created, based on scientific evidence, which contemplated guidelines including recommendations on patients’ collaborative behavior, instructions on oral hygiene, and procedures to be taken when faced with unfavorable orthodontic complications. The present study was approved by the Research Ethics Committee of *Pontifícia Universidade Católica de Minas Gerais* (CAAE #11137119.7.0000.5137).

In an attempt to make a truly appropriate content available, its validation was conducted by means of an analysis performed by a committee of specialists, Masters and Doctors in Orthodontics, members of the Brazilian Association of Orthodontics (ABOR, in Portuguese), who were lecturers or consultants in post-graduate programs in different universities. This process was based on a prior study, which reported that at least six specialists, called “judges”, should examine the following criteria:^16^

» Clarity of language: analyze if the textual content is clear, easily understandable, and appropriate for the target population.
» Theoretical relevance: consider the association between content and theory.
» Practical pertinence: evaluate if the content is appropriate for the public for which it has been created.

**Figure 1:**
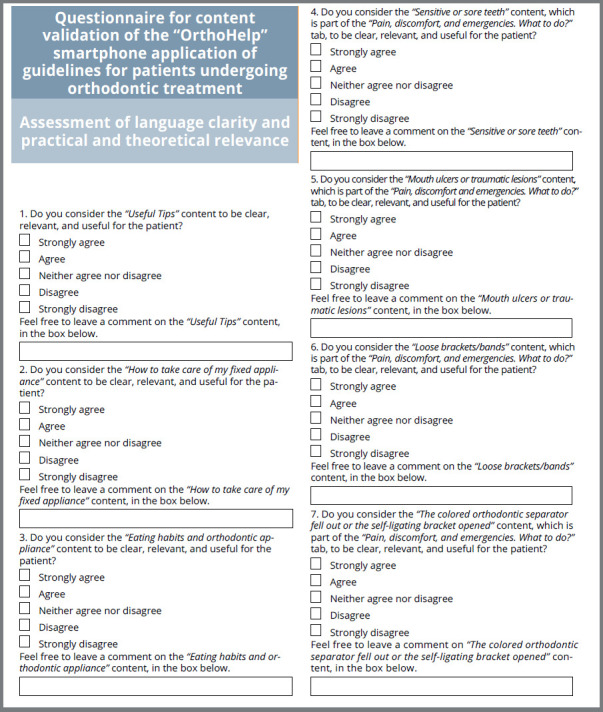
Questionnaire for content validation of the “OrthoHelp” smartphone application of guidelines for patients undergoing orthodontic treatment.

Invitation letters, together with the elaborated material (texts, images, and videos) and a virtual validation questionnaire (Fig 1), created in the online platform SurveyMonkey^®^, were sent, via e-mail, to 10 possible judges. The answers for the questionnaire were constructed following the Likert scale, with five alternatives: 1) Strongly disagree, 2) Disagree, 3) Neither agree nor disagree, 4) Agree, 5) Strongly agree. In addition to the use of this scale, a space was also created for the judges to provide criticisms, suggestions, and recommendations, which is a common procedure in validation studies.^17^ ^,^ ^18^ ^,^ ^19^ Eight judges agreed to participate in the content analysis.

Bearing in mind that the evaluation reached an average of 85% approval among the specialists (Table 1), a new validation was deemed unnecessary.^20^ The suggested changes (Table 2) were accepted, and the final content was considered to be clear, with appropriate theoretical relevance and pertinence, and appropriate for release to the target public.

**Table 1:** Average score of questions of the virtual questionnaire on Figure 1.

Questions	Average score
Number 1	88%
Number 2	84%
Number 3	84%
Number 4	80%
Number 5	88%
Number 6	84%
Number 7	88%

**Table 2:** Comments of the judges during evaluation.

Topic	Comment
Interdental toothbrush	*“Add that the use of the interdental brush must be done calmly and carefully, to avoid breaking the appliance.”*
Dental floss	*“Add 3M Super-floss.”*
Orthodontic separator	*"The photo of the orthodontic separator with the word “important” was a little confusing. The relationship between them needs a better explanation."*
Emergencies	*“Change ‘emergencies’ to ‘urgency’.”*
Useful tips	*“I would always recommend to stay in contact with the orthodontist.”*
Videos	*“Try to improve the quality of the footage.”*
Images	*“Improve picture quality.”*

From the validated material, ICT tools for Dentistry were developed, enabling the production of: one community radio program; three blog posts; four videos; and, considering the broad popularity of mobile devices with internet access, one smartphone application.

**Figure 2:**
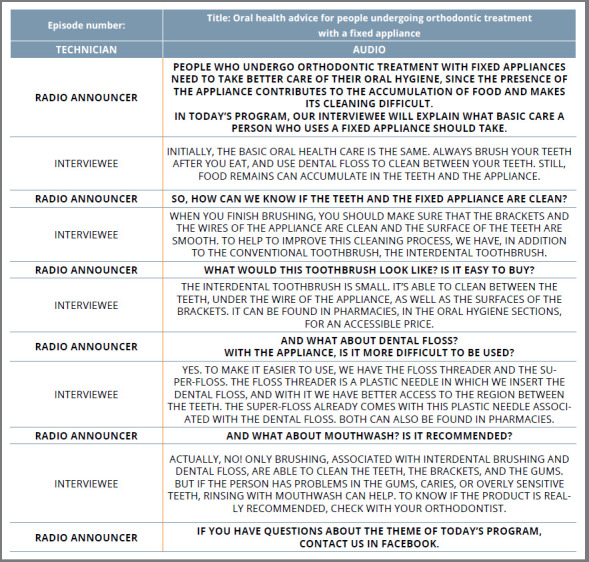
Oral health advice for people undergoing orthodontic treatment with a fixed appliance.

The community radio program scripts were drafted (Fig 2) using the information presented in the validated text. From this script, a recording was made with the researchers, answering the questions/doubts about the oral health care of patients undergoing orthodontic treatment with fixed appliances. To attend to the specificities of a blog, three texts - containing the title, a brief introduction, a short text, and a conclusion with call-to-action - were structured, resulting in three posts (advice on oral hygiene, diet, and possible urgencies). The videos were recorded using a Canon EOS Rebel T3i digital camera, and edited in the Filmora 9 Video Editor software (Wondershare Technology, HK). Four videos were developed, in order to give advice on the appropriate hygiene of teeth and of the fixed orthodontic appliance components during treatment, as well as to demonstrate how to deal with possible traumatic lesions that may take place during therapy.

A smartphone application was also developed, seeking to surpass physical and management barriers of dental care. One prototype was designed, considering functionalities, layout, and content. Among the functionalities, features such as the patient’s intuitive navigation and easy interaction were added. The layout was idealized to be “simple” and “practical”, facilitating the navigation. As regards the provided information, the validated content was organized using not only texts, but also images and videos.

The application was developed for the Android platform, which is an operational system that is highly compatible with smartphones of different brands. Tools made available by Google, such as the Android Studio development environment, were used. In addition to this environment, the Firebase development platform was also used, which is also provided by Google. The source code of the algorithms for the application was written in Java language. As user data were obtained, the SHA and MD5 algorithms (Message-Digest algorithm 5) were also used. The graphics were generated in XML (Extensible Markup Language), producing a great capacity of interaction with the user.

As the layout is the visual part of the system, menu items, buttons, and so forth were used, as is recommended by Google in its development documentation. The images and icons were generated according to the Google documentation specifications. The source project of the application was then stored in a closed repository in GitHub. Both the prototype and the development source code were registered at the National Institute of Industrial Property (INPI, in Portuguese), and the name “OrthoHelp” was chosen for the application.

During all stages of development of these technological tools, it was valued that, in addition to being reliable, the content should be accessible. In this way, all the tools were made available for free.

## RESULTS

The drafted and validated content was made available using the following ICT tools: radio, blog, videos, and smartphone application.

**Figure 3:**
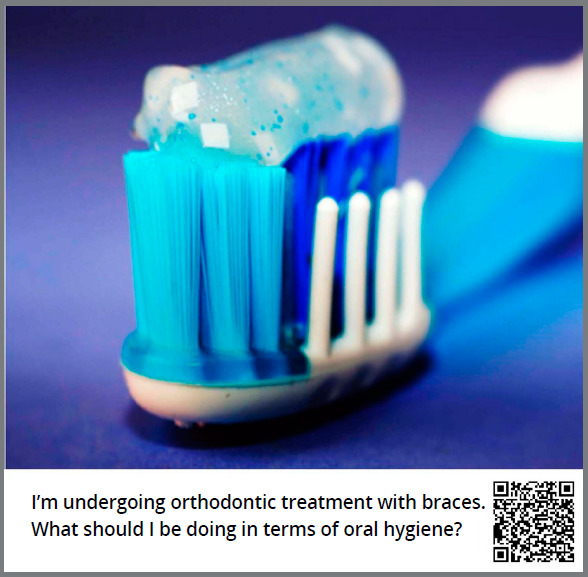
Blog post about oral hygiene advice for patients undergoing orthodontic treatment with a fixed appliance.

**Figure 4:**
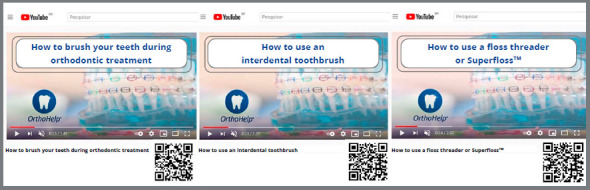
Videos about oral health care for patients undergoing orthodontic treatment with fixed appliances.

Regarding the oral health care that a patient wearing a fixed orthodontic appliance should have, dental advice concerning the correct use of the conventional toothbrush, of the interdental toothbrush, and of dental floss was offered, in an attempt to contribute to the proper cleaning of one’s teeth, as well as of the wires and brackets. Based on this theme, the following ICT tools were created: one radio program, one blog post, and three videos. The radio program, which is available for free download on the SoundCloud platform, can be reproduced by community radios that present their scheduled programs in Portuguese. The structured post for the blog received the following title: *“I’m undergoing orthodontic treatment with braces. What should I be doing in terms of oral hygiene?”* (Fig 3). And the three videos produced with this theme were: *“How to brush your teeth during orthodontic treatment”*, *“How to use the interdental toothbrush”*, and *“How to use a floss threader or Superfloss* ^*TM*^ *”* (Fig 4).

**Figure 5:**
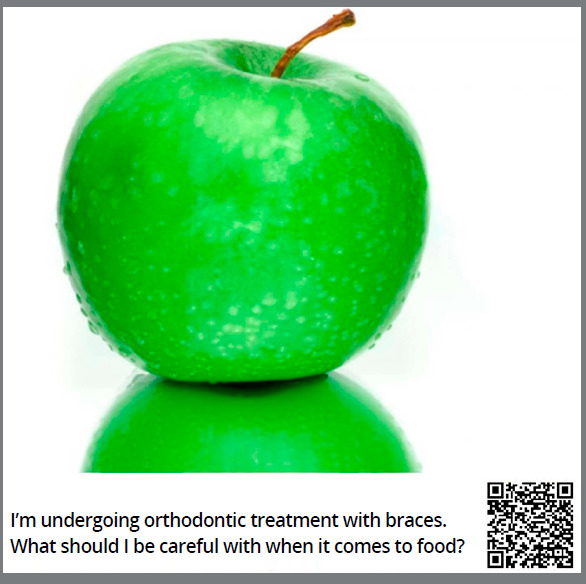
Blog post about oral health care and one’s diet for patients undergoing orthodontic treatment with fixed appliances.

As regards oral health care together with the one’s diet during orthodontic treatment with a fixed appliance, one blog post was created. The text brought information concerning the types of food that should be avoided and why (Fig 5).

**Figure 6:**
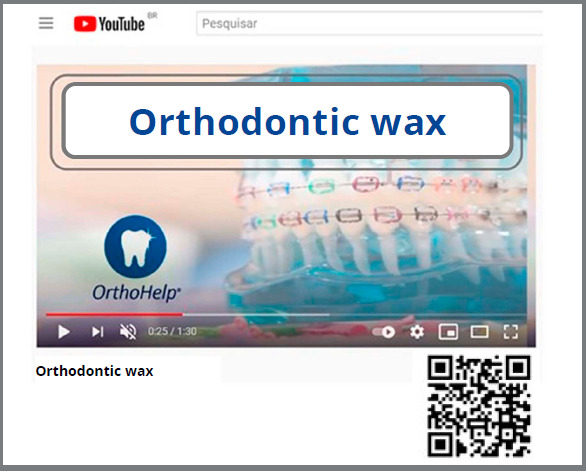
Video about the use of orthodontic wax as a preventive measure against traumatic oral lesions generated by fixed appliance accessories.

**Figure 7:**
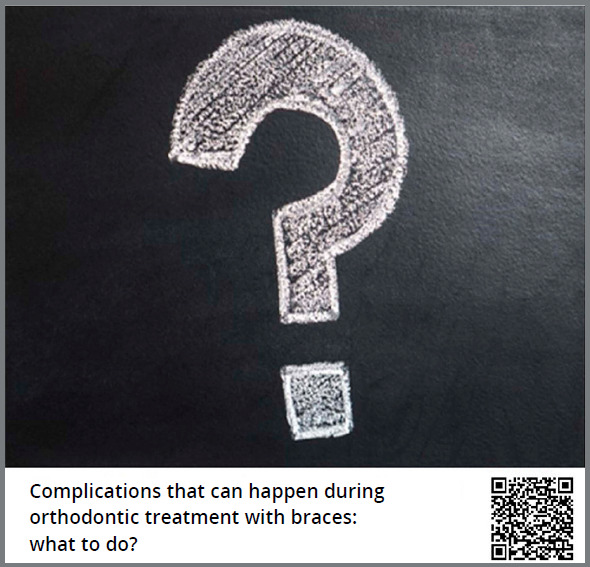
Blog post about possible complications and their respective solutions.

Regarding the possible complications that can occur during treatment and the respective provisions to be taken, one video and one blog post were made available. The video, entitled *“Orthodontic wax”*, brought information about care to be taken with traumatic lesions, presenting images and a short video that demonstrated the ease with which orthodontic wax could be used as a preventive measure against oral traumatic lesions generated by fixed appliance accessories (Fig 6). The text structured for the blog contemplated information about dental sensitivity, bracket fractures, and the detachment of orthodontic bands. Similar to the video, it also brought dental advice about the traumatic lesions that can take place due to the wearing of a fixed appliance (Fig 7).

**Figure 8:**
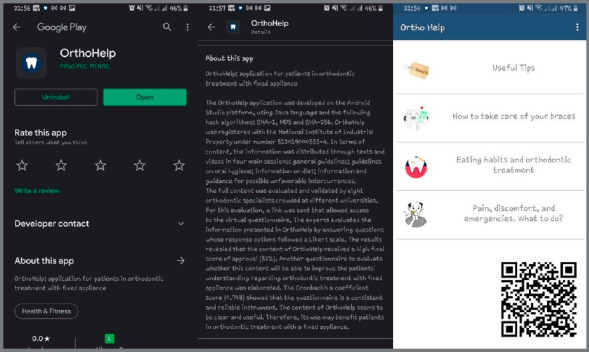
OrthoHelp smartphone application.

Considering the versatility enabled by ICTs, the OrthoHelp application was developed for smartphones. In this application, it was possible to make texts, images, and videos available. Notifications reinforcing the importance of oral health care with the fixed appliance are sent two or three times a day. The application is available for free download on Google Play, the applications store of the Android operational system (Fig 8). The application will open in English or Portuguese, according to the language option used on the smartphone.

Except for the program for community radios, which is available only for the Portuguese language, all of the other technological products are available in both English and Portuguese. Since the creation and availability of the different products, accesses, downloads and views have exceeded 450,000.

## DISCUSSION

ICTs are dynamic tools for communication and sharing information. Through these, users are able to obtain information, and express their opinions and experiences.^21^ Considering the current health scenario, these technologies have been ever-increasingly influencing the relationship between professionals and patients, offering new possibilities of work and research.^21^ Evidence, such as the increase in the number of articles published in PubMed, testing of the mentioned technologies as facilitators for the sharing of knowledge, and the social changes stemming from the adherence to such instruments, reinforce the growth of this type of contemporary approach to deal with health issues.^22^ The work developed with ICTs has adapted to this new form of how to work with science, transmitting current changes in people’s daily routines and opening the doors to contributions in the field of health care. 

The community radio sector has the support of important international communities, such as the Communication Initiative Network and the World Association of Community Radios (AMARC, in Portuguese).^5^ Examples of the relevant role that community radios play include the post-tsunami civil action in Indonesia and the Mahawweli Community Radio (MCR) in Sri Lanka, which aids displaced and landless people in resettlements,^23^ both of which are examples that reflect the promotion of democracy in communication, contributing to a more egalitarian, humane, and sustainable social development.^23^ In this sense, it was offered a community radio program destined to further self-help, to facilitate access to education and information on public health, and to aid in the promotion of a high-quality radio program geared toward patients undergoing treatment with fixed appliances.

The content was also structured to attend to the specificities of a blog. In the field of health, blogs have become quite widespread as a means of communication since 2010.^4^ In public health, they have been recognized as a quick means for sharing and exchanging experience, and to spread information to a large number of people.^4^ They are tools that allow for education to reach the target public and encourage healthy behaviors. Moreover, they must contain texts based on evidence, which are essential for a successful disclosure of health information.^6^ ^,^ ^24^ Therefore, texts written for blogs must demonstrate an adequate understanding of readers’ health needs, and provide knowledge that can be shared via reader posts and comments, creating an even broader community network, which continues beyond the blog itself.^6^ ^,^ ^25^

The use of educational videos for health promotion has been investigated.^26^ ^-^ ^29^ From the validated content, videos were created for patients undergoing orthodontic treatment with fixed appliances. Dentistry has proven to be one of the areas of health care in which the ICTs, especially videos, have been successfully applied.^15^ ^,^ ^30^ However, videos are still not efficiently verified among dental professionals.^31^ In the case of Orthodontics, a wide range of information, based on evidence or on opinions, is available in videos on YouTube^TM^.^32^ However, one prior study, evaluating videos related to Orthodontics, observed that the content made available was weak and questionable,^14^ a conclusion that was similar to another recent study^15^ reporting that, to a great extent, the reliability and quality of videos on YouTube^TM^ about the specialty are weak. More accessible information, such as oral health care during orthodontic treatment, can be easily disclosed and shared by any user on YouTube^TM^, and the fact that the information is often not provided by a dental professional confers a high probability of imprecision and unreliability to the recommendations provided.^32^ ^,^ ^33^ ^,^ ^34^ Videos on YouTube^TM^ can improve the knowledge of the patient about the orthodontic treatment, when compared with information provided in leaflets or by verbal communication, and have the advantage of being able to be accessed at any time on any mobile device with internet connection, such as smartphones.^35^ In this context, the four videos developed here expand the access and facilitate to find information about specific forms of dental care that can be done during treatment with fixed orthodontic appliances.

Mobile technology has become a common reality in daily life and has been influencing a new way of exchanging information and interactivity among its users. Smartphones and its applications have very quickly become accessible to the majority of the population, and the development of an application for mobile telephones in the field of health explores the possibility of crossing geographic and cultural barriers.^36^ ^,^ ^37^ This versatility enables information on health to be provided in a variety of formats, and it was in this context that the OrthoHelp application for smartphones was developed.

Participants of a study that evaluated preferences related to the use of applications, such as educational aids for patients undergoing orthodontic treatment, reported that the applications for dental education should be used for a better communication between dentist and patient, and to increase the patients’ understanding of the dental procedures that they are undergoing.^38^ The orthodontic complications can also be reduced and well-managed if the patients have access to concise information about the proper oral health care with the appliance through, for example, their smartphones.^1^

Notably, oral hygiene conducted in an appropriate manner can be used as an effective preventive measure against caries and to control periodontal disease and, even though it is a relatively simple action, it is constantly neglected.^39^ ^,^ ^40^ During orthodontic treatment with fixed appliances, it is possible to observe a higher degree of change in patients’ bacterial flora and an increase in the accumulation of plaque, when compared to patients who are not wearing fixed appliance.^40^ It has already been demonstrated that the use of ICTs as an aid for education in oral health has improved the periodontal indices of individuals undergoing orthodontic treatment.^10^ ^,^ ^39^

ICTs are widely used in health, even among the less favored social classes, and faced with this social relevance, the dissemination of reliable and precise information in oral health care needs to be encouraged and expanded.^41^ Therefore, in the present study, content validated by specialists, which largely contemplated oral health care advice and possible complications, provided the users appropriate and safe information regarding these important aspects during orthodontic treatment with fixed appliances. 

## CONCLUSIONS

Technological products, provided at no cost to the users through specific free ICT tools, make it possible to facilitate the access to scientific knowledge, which is of utmost importance, especially in terms of social inclusion. In addition, they provide alternative and complementary means of education and encouragement of health care, which can contribute to the quality of life of patients undergoing treatment with fixed orthodontic appliances.

By providing a wide range of technical tools created in this study (radio program, blog posts, videos, and smartphone application), it is our intent to contribute to advances in the concession of information to the general population, fact that can be corroborated by the large number of accesses, downloads and views (which already exceed 450,000) to the content developed here. These tools can favor the patient’s understanding of the explanations provided by dentists, which can instill a greater peace of mind, security, and comfort during orthodontic therapy for both patients and dental professionals.
